# Self-efficacy and fatigue among non-frontline health care workers during COVID-19 outbreak: A moderated mediation model of posttraumatic stress disorder symptoms and negative coping

**DOI:** 10.1371/journal.pone.0243884

**Published:** 2020-12-10

**Authors:** Tianya Hou, Ruike Zhang, Xiangrui Song, Fan Zhang, Wenpeng Cai, Ying Liu, Wei Dong, Guanghui Deng

**Affiliations:** Faculty of Psychology, Second Military Medical University, Shanghai, China; University of British Columbia, CANADA

## Abstract

**Purposes:**

Since a considerable number of health care workers (HCWs) were sent to Wuhan to aid COVID-19 control during the epidemic, non-frontline HCWs who stayed in local hospitals had to work overload to provide daily health care services for other health issues, which makes them more vulnerable to experience fatigue. Self-efficacy is suggested as a protective factor for fatigue. Nonetheless, less is known regarding the underlying mechanisms. This research aimed to explore the prevalence of fatigue among non-frontline HCWs during the pandemic, investigate the mediating effect of posttraumatic stress disorder (PTSD) symptoms and moderating effect of negative coping in the association between self-efficacy and fatigue.

**Methods:**

General Self-Efficacy Scale, PTSD Checklist-Civilian Version, Simplified Coping Style Questionnaire and 14-item Fatigue Scale were administrated to 527 non-frontline HCWs from Anhui Province, China. The mediating effect was examined by Mackinnon’s four-step procedure, while Hayes PROCESS macro was used to test the moderated mediation model.

**Results:**

The prevalence of fatigue among non-frontline HCWs was 56.7%. The effect of self-efficacy on fatigue was partially mediated by PTSD symptoms (ab = -0.146, SE = 0.030, 95% CI = [-0.207, -0.095]). Additionally, negative coping moderated both the direct effect of self-efficacy on fatigue (*β* = -0.158, *P<*0.001) and the mediating effect of PTSD symptoms (*β* = 0.077, *P* = 0.008). When the standard score of negative coping increased to 1.49 and over, the direct association between self-efficacy and fatigue became insignificant. Likewise, the effect of self-efficacy on PTSD symptoms had no statistical significance when the standard score of negative coping was -1.40 and lower.

**Conclusions:**

More than half non-frontline HCWs suffered from fatigue during COVID-19. For those who tend to use negative coping, it might be crucial to design programs combining the enhancement of self-efficacy, preventions for PTSD symptoms and interventions for fatigue.

## Introduction

On the last day of 2019, Coronavirus Disease 2019 (COVID-19) with unknown etiology was first reported in Wuhan, China [1]. On 30 January, 2020, the COVID-19 outbreak was declared a Public Health Emergency of International Concern [2]. The outbreak as a global health threat rapidly spread [3]. As of July 18, 2020, more than 13 million confirmed cases have been reported from almost every country and a near exponential growth in the number of new confirmed cases has been witnessed over the past few weeks [4]. The COVID-19 epidemic is straining health care systems with escalating demand on health care workers (HCWs) and health facilities. The availability of professional HCWs would largely determine whether the pandemic could be defeated [5] and the Chinese government rapidly mobilized a large number of HCWs nationwide to support the epicenter [6], resulting in more than 42,000 HCWs from other provinces in Wuhan by the end of February [7]. Recent COVID-19 studies presented non-frontline HCWs had more mental health problems than frontline HCWs due to less first-hand information, insufficient psychological support, lack of medical resources and great pressure to provide daily health care services and maintain regular treatment for other health issues [8, 9]. Given that the majority of the studies about the psychological impact of COVID-19 are about frontline HCWs, more attention should be paid to non-frontline HCWs, which is of great importance to maintain and enhance the efficiency, quality and safety in the health sector amid COVID-19 outbreak.

Work-related fatigue, as a longstanding problem in health care settings, is associated with reduced vigilance and poor work performance [10], increasing the incidence of medical errors and jeopardizing work efficiency and quality. Considerable HCWs have been sent to Wuhan during the COVID-19 pandemic, resulting in the shortage of HCWs in local hospitals in other regions. To maintain daily health care services, non-frontline HCWs need to work overload and face numerous stressors, which makes them more vulnerable to experience fatigue [11]. Hence, there is an urgent demand on investigation of the influential factors and underlying mechanisms of fatigue to design targeted interventions against fatigue.

Fatigue, as a multidimensional state, could be caused by numerous factors, which makes the identification of underlying mechanisms challenging [12]. A two-stage approach to manage fatigue has been proposed. The first stage is to deal with treatable factors, while the second stage is to address residual fatigue. Fatigue has been widely studied in clinical samples and self-efficacy has been identified as one of the influential nonpharmacological factors [13, 14]. Self-efficacy is defined as the belief of one’s capacity to successfully accomplish specific goals [15]. Numerous studies have suggested the enhancement of self-efficacy could be particularly effective in ameliorating fatigue [16] since it could protect against the adverse influence of stressors [17]. However, there is a lack of knowledge regarding the association between self-efficacy and fatigue among non-frontline HCWs during the pandemic. In addition, the mechanisms behind the association are not well-understood.

The COVID-19 epidemic is a continuing crisis for everyone in the society [18], and it is well-known that public health emergency could exert an adverse mental health impact on various groups (such as HCWs, patients and general public) [19–21], leading to depression, anxiety, insomnia and posttraumatic stress disorder (PTSD) [22–24]. Thus, non-frontline HCWs are also vulnerable to develop PTSD, a psychiatry disorder caused by the witness or experience of traumatic events, due to sympathy for patients, concern about colleagues who went to Wuhan and fear of being infected during COVID-19 [25, 26]. The most common PTSD symptoms are recurrent memory about traumatic events, avoidance and heightened arousal [27]. An extensive body of literature showed general self-efficacy was a significant predictor of PTSD symptom [28, 29]. Previous literature demonstrated high self-efficacy participants showed less distress after the trauma film paradigm in comparison to those from low self-efficacy group [30]. A recent study conducted by Titcombe-Parekh et al. suggested the increase in self-efficacy could impact neural circuits regarding executive function and emotional regulation, which would further contribute to the decrease of PTSD symptoms [31]. More importantly, the findings from a prospective longitudinal study presented self-efficacy was negatively associated with subsequent PTSD symptoms over an 8-year follow-up period [32]. Furthermore, another recent research based on a sample of civilian war victims reported hyper-arousal and active avoidance symptoms of PTSD mediated the relation between the exposure to trauma and somatic symptoms such as fatigue since PTSD symptoms might lead to enhancing muscle tension, increasing alertness of pain and negative appraisals towards experience [33, 34]. Thus, it is possible that PTSD symptoms would mediate the association between self-efficacy and fatigue of non-frontline HCWs during the outbreak.

Coping style is defined as the thoughts or behaviors an individual adopts to handle the adversity and stress [35]. Previous literature did not reach a consensus concerning the classification of coping style [36, 37]. Lazarus and Folkman suggested coping styles could be categorized into emotion-focused and problem-focused coping [35]. The former focuses on reducing negative emotions, including strategies such as blaming and avoidance, while the latter aims to manage problems, such as problem solving and seeking social support. Some researchers also divided coping into engagement and disengagement, with the former dealing with stressors or negative emotions and the latter avoiding stressful events or related feelings [38]. It could be found that no matter how coping is classified, some coping styles are more positive, while others are more negative. Thus, coping styles could also be classified into negative coping and positive coping [39]. Negative coping involves denial, avoidance, wishful thinking and withdrawal, while positive coping refers to solving problems in a rational and direct way [40]. An emerging body of studies provided evidence regarding the interaction effect of self-efficacy and coping style [41], indicating the effect of self-efficacy might be influenced by coping style. According to the integrative framework of coping process, self-efficacy as a personal factor and coping style as a transactional-situational process are interrelated to determine health outcomes [42]. Findings from Witkiewitz and Marlatt presented low self-efficacy was a part of a person’s vulnerability to psychological problems and negative coping could aggrevate this vulnerability [43]. Levin et al. [44] proposed avoidance coping moderated the effect of self-efficacy on alcohol use outcomes at 5 years. All these suggest a moderating model and support the hypothesis that coping might influence the effect of self-efficacy on health outcomes. Nonetheless, it remains unexplored whether coping style moderated the effect of self-efficacy on PTSS and fatigue among non-frontline HCWs during the epidemic. Also, previous findings suggested avoidant coping moderated the effect of self-efficacy on health outcomes, whereas positive coping failed to moderate the association [44]. Moreover, previous literature presented consistent results in terms of the relationship between negative coping and health outcomes and the inconsistent results concerning the effect of positive coping on health outcome since the effectiveness of positive coping might be a more crucial determinant in the positive outcome [45, 46]. Therefore, the current study would only focus on the moderating effect of negative coping as there was no method to measure the effectiveness of positive coping during COVID-19.

### The present study

**Fig 1:**
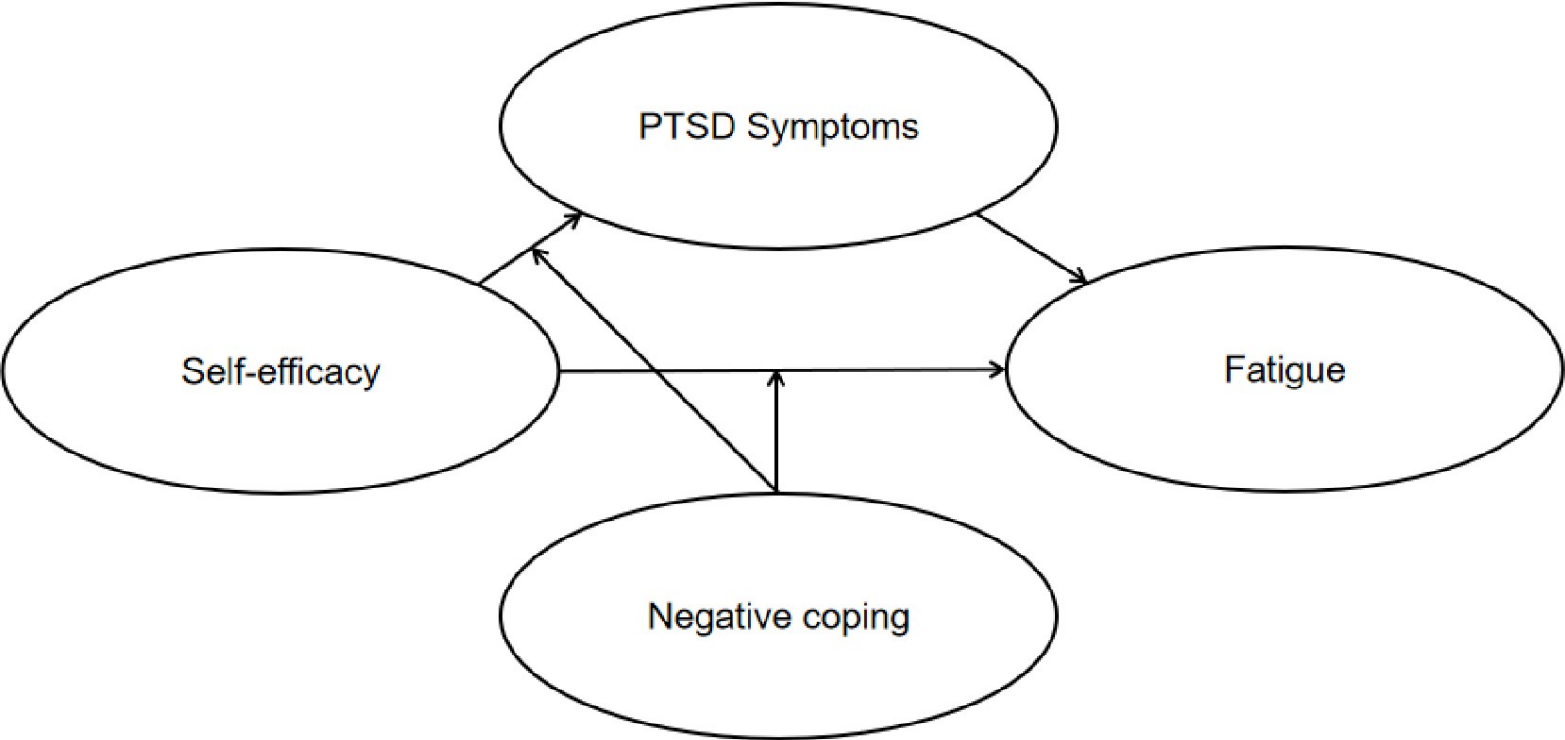
The schematic model of proposed moderated mediation model.

In sum, the current study aimed to explore the prevalence of fatigue among non-frontline HCWs during the outbreak of COVID-19, and investigate the mediating role of PTSD symptoms and the moderating role of negative coping in the association between self-efficacy and fatigue. Thus, we proposed a moderated mediation model (see Fig 1) to address the hypotheses that PTSD symptoms might mediate the effect of self-efficacy on fatigue and negative coping might moderate the direct and/or indirect (self-efficacy–PTSD symptoms path) effect of self-efficacy on fatigue among non-frontline HCWs during the COVID-19 pandemic. This study could provide a theoretically grounded foundation for an in-depth understanding of fatigue and its influential factors among non-frontline HCWs and inform public health policy of potential preventive interventions.

## Methods

### Participants

This cross-sectional survey was performed in Anhui Province, China. The region borders Hubei province, the epicenter of the COVID-19 outbreak. All data were collected between March 13–20, 2020, more than 2 months after the outbreak of COVID-19 in China. A total of 528 HCWs from 5 hospitals in Anhui province were selected using random cluster sampling. The inclusion criteria were a) working in the hospital during the pandemic, b) age > 18. The exclusion criterion was that HCWs who had direct contact with COVID-19 infected patients. Finally, 527 subjects were included in the analysis (effective response rate 99.8%). All participants in our analysis were physicians or nurses. The study was approved by the research ethics committee of Second Military Medical University. Before filling out the online questionnaires, informed written consent was obtained from each participating subject. To protect participants’ privacy and encourage honest reporting, the questionnaires were finished anonymously. In addition, participants were told the participation was voluntary and they could withdraw at any time.

### Measures

#### Self-efficacy

The Chinese version of the General Self-Efficacy Scale (GSES) developed by Zhang and Schwarzer [47] was used to measure self-efficacy. The 10-item scale only included one dimension and each item was scored on a 4-point Likert scale from 1 (not true at all) to 4 (exactly true). The range of the total scores was 10–40, with higher scores indicating higher levels of self-efficacy. The scale has been demonstrated with good construct validity, impressive test-retest reliability and excellent internal consistency in Chinese samples [47, 48]. In the present study, the Cronbach’s Alpha for GSES was 0.900.

#### PTSD symptoms

The 17-item PTSD Checklist-Civilian Version (PCL-C) with three subscales (re-experiencing, avoidance and hyperarousal) was used to assess PTSD symptoms with regard to COVID-19 [49]. Each item was rated on a 5-point Likert scale ranging from 1 (not at all) to 5 (extremely). The 17 items were summed to create a total score representing the severity of PTSD symptoms, with higher scores denoting more severe PTSD symptoms. The Chinese version of the scale has presented high internal consistency and adequate convergent validity [50]. The Cronbach’s Alpha in the present study for PCL-C was 0.963.

#### Negative coping

To assess the degree of negative coping, we adopted the 8-item negative coping subscale of Simplified Coping Style Questionnaire (SCSQ) [51]. Each item was rated on a 4-point Likert scale ranging from 0 = never used to 3 = often used (e.g., “Rely on others to solve problems”) and they were averaged to indicate the tendency to use negative coping, with higher scores representing a greater tendency to use negative coping. The negative coping subscale has shown good internal consistency and test-retest reliability [51]. In the current study, the Cronbach’s Alpha for the negative coping subscale of SCSQ was 0.804.

#### Fatigue

The 14-item Fatigue Scale (FS-14) was employed to evaluate fatigue in the past week [52]. Each item described a symptom relevant to fatigue (e.g., “Do you feel weak?”). Participants rated each item with two responses: 0 (no symptom) and 1 (having symptoms). The total score was 0–14 points. According to the previous literature based on Chinses samples [53], the threshold considered for detecting fatigue was a score of 7 or above on FS-14. The Chinese version of the scale has been widely used in health care settings with good validity and reliability [11, 54]. In the study, the Cronbach’s Alpha for FS-14 was 0.907.

#### Covariates

In the current study, the covariates included age, gender, marital status, educational level, years of working and technical title. Age was grouped into 20–29 years, 30–39 years, 40–49 years and 50–59 years. Marital status was divided into unmarried (single, divorced and widowed) and married. Educational level was categorized into two groups: high school or under and university or above. Years of working was divided into 10 years or less and more than 10 years. Technical title was classified into three groups: junior, intermediate and senior.

### Statistical analysis

Firstly, we used descriptive analyses to describe demographic and working characteristics. Independent t-test and one-way analysis of variance (ANOVA) followed by LSD post hoc test were used to compare group differences in fatigue. Secondly, bivariate correlations between all the study variables (self-efficacy, PTSD symptoms, negative coping and fatigue) were calculated by Pearson’s correlation analyses. Thirdly, the mediation effect was examined according to Mackinnon’s four-step procedure [55]. Four conditions need to be met: (1) a significant association between self-efficacy and fatigue; (2) a significant relationship between self-efficacy and PTSD symptoms; (3) a significant relationship between PTSD symptoms and fatigue while controlling for self-efficacy; (4) a significant coefficient for the indirect association between self-efficacy and fatigue via PTSD symptoms. The last condition was examined by the bias-corrected percentile bootstrap method [56], producing a 95% bias-corrected confidence interval (CI) with 5000 replacements. The effect would be determined if the 95% CI does not include 0. Hayes PROCESS macro (Model 4) [56] was employed to estimate parameters for the mediation effect.

Finally, the moderated mediation effect was examined by Model 8 [56]. As mentioned above, the effects were established if the 95% bias-corrected bootstrap CI of the interaction excluded 0. Then, Johnson-Neyman technique [57] was employed to plot the conditional effects and confidence bands at different values of negative coping. In addition, z-scores for each variable were calculated before the analysis. Furthermore, all models were controlled for age, gender, marital status, educational level, years of working and technical title. All statistical analyses were performed by SPSS 25.0 and two-tailed *P*-values less than 0.05 were regarded as statistical significance.

## Results

### Demographic and working characteristics and fatigue

The characteristics of the sample and the group comparisons on fatigue are presented in Table 1. Most non-frontline HCWs were female (65.3%) and married (80.8), obtained the degree of university or above (67.2%), and worked 10 years or less. The mean age of 527 non-frontline HCWs was 34.86 (SD = 8.67), ranging from 20 to 58 with 294 (55.8%), 178 (33.8), 55 (10.4) respondents reporting junior, intermediate and senior technical title, respectively.

**Table 1:** Demographic and working characteristics of respondents (*N* = 527) and group comparisons on fatigue.

	Respondents		Fatigue Scores		*F/t*	*P*-value
	*n*	%	M	SD		
Gender					1.489	0.223
Male	183	34.7	7.14	4.43		
Female	344	65.3	7.64	4.51		
Marital status					1.425	0.233
Unmarried	101	19.2	6.99	4.64		
Married	426	80.8	7.58	4.44		
Educational level					0.108	0.742
High school or below	173	32.8	7.56	4.41		
University or above	354	67.2	7.42	4.52		
Years of working					0.714	0.398
10 years or less	272	51.6	7.31	4.61		
More than 10 years	255	48.4	7.64	4.35		
Age (mean = 34.86, SD = 8.67)					3.176	0.024
20–29	175	33.2	7.06	4.63		
30–39	213	40.4	8.16	4.46		
40–49	88	16.7	7.16	4.31		
50–59	51	9.7	6.49	4.09		
Technical title					2.233	0.108
Junior	294	55.8	7.15	4.59		
Intermediate	178	33.8	8.04	4.30		
Senior	55	10.4	7.35	4.36		

The prevalence of fatigue among non-frontline HCWs was 56.7% (FS-14 ≥ 7). There was a significant difference in fatigue among different age groups (*F* = 3.176, *P* = 0.024). LSD *post hoc* test indicated non-frontline HCWs aged 30–39 years presented significant higher levels of fatigue than those aged 20–29 years and those aged 50–59 years (all *P* < 0.05). No significant differences were found in fatigue by gender, marital status, educational level, years of working and technical title (all *P* > 0.05).

### Bivariate correlations between all the study variables

As presented in Table 2, self-efficacy was negatively correlated with PTSD symptoms (*r* = -0.301, *P* < 0.001) and fatigue (*r* = -0.402, *P* < 0.001). PTSD symptoms were positively associated with negative coping (*r* = -0.336, *P* < 0.001) and fatigue (*r* = -0.402, *P* < 0.001). In addition, negative coping was positively related to fatigue (r = 0.143, *P* < 0.01). However, there was no significant association between self-efficacy and negative coping (*P* > 0.05).

**Table 2:** Pearson’s correlation among self-efficacy, PTSD symptoms, negative coping and fatigue (*N* = 527).

	M	SD	1	2	3
Self-efficacy	26.34	4.24			
PTSD symptoms	31.25	12.52	-0.301***		
Negative coping	1.15	0.55	0.040	0.336***	
Fatigue	7.47	4.48	-0.402***	0.576***	0.143**

** *P* < 0.01

*** *P* < 0.001

### Mediating effect of PTSD symptoms

The study assumed PTSD symptoms would mediate the relationship between self-efficacy and fatigue. We followed Mackinnon’s four-step procedure to examine the mediation effect (see Table 3). Firstly, self-efficacy was significantly associated with fatigue (*β* = -0.392, *P<*0.001) (see Model 1 in Table 3). Secondly, self-efficacy was significantly related to PTSD symptoms (*β* = -0.296, *P<*0.001) (see Model 2 in Table 3). Thirdly, PTSD symptoms were significantly correlated with fatigue when we controlled for self-efficacy (*β* = 0.493, *P<*0.001) (see Model 3 in Table 3). Finally, the indirect effect of self-efficacy on fatigue via PTSD symptoms was significant (ab = -0.146, SE = 0.030, 95% CI = [-0.207, -0.095]). The mediation effect accounted for 37.2% of the total effect. In sum, all four criteria for mediation effect have been met and PTSS symptoms mediated the effect of self-efficacy on fatigue of non-frontline HCWs during the COVID-19 pandemic.

**Table 3:** Mediation analysis (*N* = 527).

	Model 1(Fatigue)		Model 2 (PTSD symptoms)		Model 3(Fatigue)	
	*β*	*t*	*β*	*t*	*β*	*t*
Self-efficacy	-0.392***	-9.589	-0.296***	-6.972	-0.246***	-6.692
PTSD symptoms					0.493***	13.533
R^2^_adj_	0.163***		0.095***		0.381***	
F	11.247		6.530		30.485	

*Note*: All models are adjusted for age, gender, marital status, educational level, years of working and technical title.

***P<0.001

### Moderated mediation effect analysis

The study anticipated negative coping might play as a moderator in the direct and indirect (the first stage of the mediation pathway: self-efficacy–PTSD symptoms) effects of self-efficacy on fatigue. As presented in Table 4, the results of moderated mediation analysis showed the interaction of self-efficacy and negative coping had a significant effect on PTSD symptoms (*β* = -0.158, *P<*0.001), indicating the association between self-efficacy and PTSD symptoms was moderated by negative coping. The moderated mediation effect was established since the indirect pathway was moderated by negative coping [56]. Additionally, negative coping also moderated the direct effect of self-efficacy on fatigue (*β* = 0.077, *P* = 0.008).

**Table 4:** Conditional process analysis (*N* = 527).

	*β*	*SE*	LLCI	ULCI
Mediator variable model (Outcome: PTSD symptoms)				
Self-efficacy	-0.326***	0.039	-0.402	-0.249
Negative coping	0.363***	0.038	0.287	0.438
Self-efficacy * Negative coping	-0.158***	0.031	-0.219	-0.097
Dependent variable model (Outcome: Fatigue)				
Self-efficacy	-0.228***	0.037	-0.302	-0.154
PTSS symptoms	0.521***	0.040	0.443	0.600
Negative coping	-0.030	0.038	-0.104	0.044
Self-efficacy * Negative coping	0.077**	0.029	0.020	0.134
	*β*	Boot *SE*	BootLLCI	BootULCI
Conditional direct effect analysis				
1 SD below the mean	-0.305	0.044	-0.390	-0.220
Mean	-0.228	0.037	-0.302	-0.154
1 SD above the mean	-0.151	0.051	-0.251	-0.051
Conditional indirect effect analysis				
1 SD below the mean	-0.087	0.023	-0.138	-0.047
Mean	-0.170	0.025	-0.222	-0.123
1 SD above the mean	-0.252	0.037	-0.328	-0.183
Index of moderated mediation	-0.082	0.017	-0.118	-0.048

*Note*: All models are adjusted for age, gender, marital status, educational level, years of working and technical title.

* *P* < 0.05

*** *P* < 0.001

**Fig 2:**
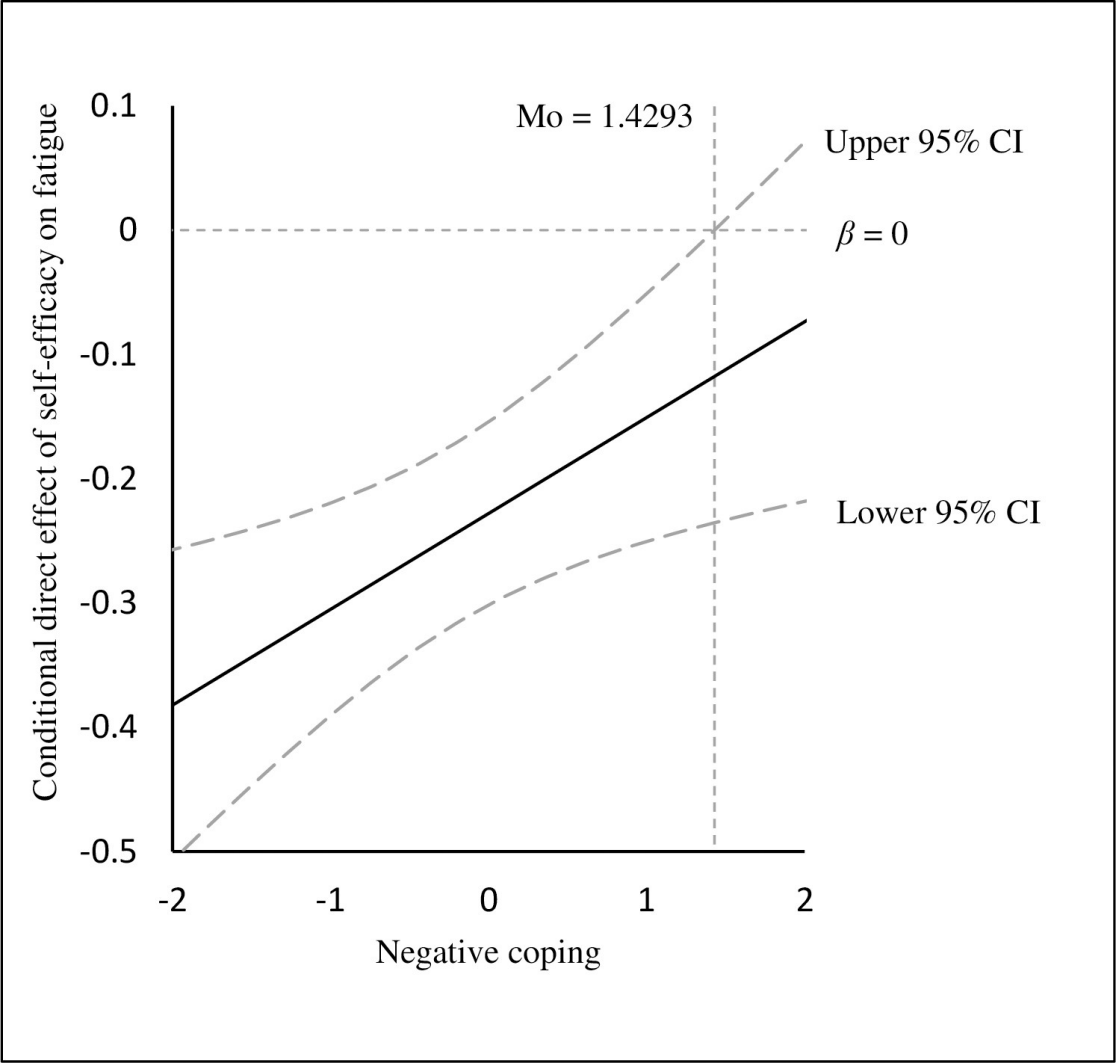
The conditional direct effect of self-efficacy on fatigue at the values of negative coping.

Table 4 also showed the conditional direct and indirect effects of self-efficacy on fatigue at different values of negative coping (1 SD below the mean, the mean, and 1SD above the mean). The direct effect of self-efficacy on fatigue was stronger at 1 SD below the mean of negative coping (*β* = -0.305, 95% CI: -0.390, -0.220) than 1SD above the mean (*β* = -0.151, 95% CI: -0.251, -0.051). As shown by Johnson-Neyman technique [57], negative coping would moderate the direct effect of self-efficacy on fatigue when the standard scores of negative coping were lower than 1.429, in which the 95% CI did not contain zero (see Fig 2).

**Fig 3:**
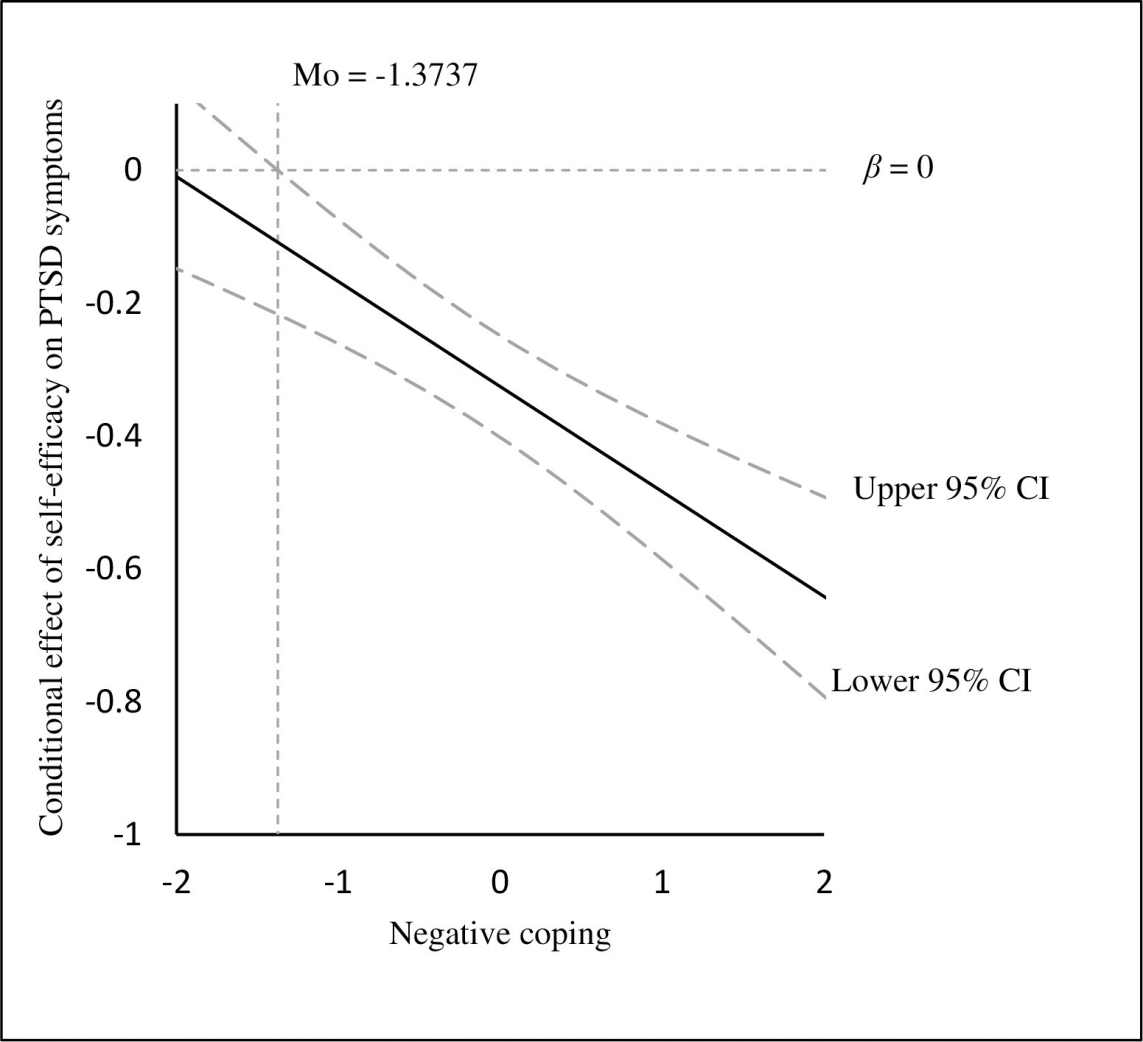
The conditional effect of self-efficacy on PTSD symptoms at the values of negative coping.

Nonetheless, the indirect effect of self-efficacy on fatigue through PTSD symptoms was attenuated at 1 SD below the mean of negative coping (*β* = -0.087, 95% CI: -0.138, -0.047) in comparison to 1 SD above the mean (*β* = -0.252, 95% CI: -0.328, -0.183). Johnson-Neyman technique presented negative coping would moderate the association between self-efficacy and PTSD symptoms when the standard scores of negative coping were more than -1.374 as the 95% CI did not include zero (see Fig 3).

## Discussion

To the best of our knowledge, this is the first study to investigate the prevalence of fatigue among non-frontline HCWs during the COVID-19 epidemic and explore the potential mechanisms underlying the association between self-efficacy and fatigue with PTSD symptoms and negative coping as the mediator and moderator, respectively.

The prevalence of fatigue among non-frontline HCWs was 56.7%, which is higher than a systematic review summarizing the prevalence of fatigue among HCWs during COVID-19 with an overall incidence rate of fatigue among paramedics of 38% [58]. Consistent with the previous literature [8, 9], our findings suggested non-frontline HCWs appeared to be more susceptible to fatigue than frontline HCWs during the outbreak. Interestingly, when it comes to the comparison with the prevalence of fatigue among HCWs in the non-epidemic period, the results were inconsistent. Considerable studies found the lower prevalence of fatigue ranging from 21.6 to 45.5% [59–62], whereas Da Silva et al. [63] reported the overall incidence rate of fatigue among nursing workers in Brazil was 52%, which is in line with our findings. Moreover, several research observed higher incidence rates of fatigue ranging from 83.7 to 91.9% [54, 64, 65]. The discrepancy might be attributed to different definitions of fatigue, diverse assessment tools, inconsistent cut-off points and so forth. For instance, Cai et al. [66] employed a score of 4 on a 11-item fatigue scales as the cut-off point to define the occurrence of fatigue, while O'Donnell et al. [62] measured fatigue through only one question (the self-assessment of the average level of fatigue during the previous week). These differences might account for the different prevalences of fatigue among HCWs. However, there is no doubt that fatigue is a commonly experienced symptom among non-frontline HCWs during the COVID-19 outbreak and more attention should be paid to deal with this issue to maintain the work safety and efficiency in the health care settings.

Our results found there were significant differences in fatigue among different age groups. Specifically, congruent with the previous literature [61, 66], the 30–39 years group reported significant higher levels of fatigue in comparison to the age groups of 20–29 and 50–49 years. The older hospital staff with richer working experience and stronger professional skills usually worked as group leaders to make decisions, whereas those aged 30–39 years implemented the decisions with physical labor. In addition, the non-frontline HCWs from 30–39 years group took more responsibilities than those aged 20–29 years since the younger hospital staff might lack experience and sufficient professional knowledge and could not complete the work alone. Furthermore, those aged 30–39 are more likely to face the responsibilities to provide support and care to both young children and elderly parents [67, 68], which could further result in the higher levels of fatigue. Therefore, those reasons might explain the differences. In the current study, consistent with some previous literature [54], there was no gender difference in fatigue. However, several previous studies claimed women were more likely to suffer from fatigue [11, 64]. The inconsistent results might be attributed to the socio-economic status of participants. As Jenkins proposed [69], when controlling for socio-economic backgrounds, the gender difference in the rates of minor psychiatric morbidity would disappear. In addition, one of the genders might be under-represented in some studies [70], which might partially explain the difference.

In line with our hypothesis, this study demonstrated a partially mediating role of PTSD symptoms in the association between self-efficacy and fatigue, suggesting the potential mechanisms regarding how self-efficacy would indirectly affect fatigue. Low self-efficacy could not only directly contribute to higher levels of fatigue, but also indirectly aggravate fatigue via PTSD symptoms. This is consistent with the previous literature during SARS outbreak in terms of the mediating role of posttraumatic stress scores in the association between the risk of exposure and perceived stress among HCWs [71]. This study extended the previous literature by combining self-efficacy as a protective factor and PTSD symptoms as a risk factor to explore fatigue, which has profound implications for the prevention and mitigation of fatigue of non-frontline HCWs during COVID-19. The self-efficacy-based program and intervention for PTSD could be designed to reduce the occurrence of fatigue during the COVID-19 pandemic, which might further decrease the medical errors and enhance the work quality.

More importantly, the moderated mediation analysis presented negative coping could moderate the direct and indirect effects of self-efficacy on fatigue of non-frontline HCWs. This is consistent with the integrative framework of coping behaviors and the previous study [42, 44], suggesting the moderating role of coping style in the link between self-efficacy and health outcomes. As revealed by Johnson-Neyman technique, it is noteworthy the direct effect of self-efficacy on fatigue became weakened with increasing negative coping. When the standard score of negative coping enhanced to 1.49 and over, the direct association was not significant any more. In contrast, the indirect effect of self-efficacy on fatigue via PTSD symptoms strengthened as the level of negative coping increased. Likewise, Johnson-Neyman technique showed the effect of self-efficacy on PTSD symptoms had no statistical significance when the standard score of negative coping was -1.40 and lower. This adds to our understandings of fatigue with important practical implications. Interventions for PTSD should be prioritized for non-frontline HCWs who tend to use negative coping as self-efficacy would be more likely to influence fatigue through PTSD symptoms.

Our focus, in the current conceptual model, is to explore and mitigate fatigue of non-frontline HCWs. However, our results may be an artifact of reverse causality and the direction of the association between self-efficacy and health outcomes cannot be determined due to the cross-sectional design. Our supplementary analysis also presented both PTSD symptoms and fatigue could predict self-efficacy after controlling for demographic variables (see S1 Appendix). This indicates the possibility that non-frontline HCWs’ belief in their ability to successfully perform a task might be shattered by the PTSD symptoms and fatigue they experienced during the COVID-19 pandemic [32, 72]. The interventions to manage fatigue such as increasing social support, enhancing sleep quality and reducing extended work shifts should also be highlighted as complementary strategies during the pandemic [70, 73].

Several limitations should be addressed. Firstly, the present study employed a cross-sectional design, which cannot verify the temporal sequence of the variables. It is difficult to tease apart the cause-and-effect relationships. It is just as likely that fatigue could lead to reduced self-efficacy and increased negative coping and PTSD symptoms as the direction proposed in our study. It remains elusive whether self-efficacy influences PTSD symptoms and fatigue or vice versa or whether they affect each other mutually. The longitudinal or experimental studies should be conducted to further explore the associations. Secondly, the data were obtained through self-report questionnaires, which might cause self-reported biases. Further study could collect data from diverse informants. Thirdly, the participants were only from Anhui province, limiting the generalization of the results to other areas. Further study would recruit subjects from diverse regions. Fourthly, the demographic information including occupation and hospital working unit might influence fatigue, PTSD symptoms and coping. However, these data were not collected in the present study to achieve anonymity and more advanced confidentiality, which might have slightly biased the results. Fifthly, negative coping was found to moderate the effect of self-efficacy on health outcomes in our study, whereas previous literature has also indicated coping could mediate the effect of self-efficacy on disability [74]. Better understanding of the relationship between self-efficacy and coping, which exerted an impact on health outcomes, is in need to further provide guidance for a better practice. Finally, as mentioned above, fatigue could be influenced by many factors [12]. Our model could just explain a part of the variance. A more integrative model is suggested for the future study.

## Conclusions

The prevalence of fatigue among non-frontline HCWs during the COVID-19 outbreak was 56.7% in the study. PTSD symptoms partially mediated the effect of self-efficacy on fatigue. In addition, both the direct effect of self-efficacy on fatigue and the mediating effect of PTSD symptoms were moderated by negative coping. Specifically, the direct effect was weaker and the indirect effect was stronger for non-frontline HCWs who were more likely to adopt negative coping. For non-frontline HCWs, especially those who were more likely to use negative coping, it might be of vital importance to design programs combining the enhancement of self-efficacy and preventions for PTSD to mitigate fatigue. In the meantime, the interventions for fatigue should be employed as complementary strategies during the epidemic.

## Supporting information

S1 Appendix The effect of PYSD symptoms and fatigue on self-efficacy. (DOCX)

S1 Dataset The minimal anonymized data set necessary to replicate study findings. (SAV)
