# Supplementary material for: Self-efficacy and fatigue among non-frontline health care workers during COVID-19 outbreak: A moderated mediation model of posttraumatic stress disorder symptoms and negative coping
Source: PLoS One. 2020 Dec 10;15(12):e0243884. doi: 10.1371/journal.pone.0243884 (PMC7728176; doi:10.1371/journal.pone.0243884)
Supplement: S1 Appendix — (DOCX) [file pone.0243884.s001.docx]

**S1 Appendix. The effect of PYSD symptoms and fatigue on self-efficacy.**

The supplementary analysis presented in S1 Table investigated the effect of PTSD symptoms and fatigue on self-efficacy among non-frontline health care workers during the COVID-19 pandemic. When we controlled for covariates, both PTSD symptoms and fatigue were significantly correlated with self-efficacy (*β* = -0.107, *P*＜0.05 and *β* = -0.330, *P*＜0.001, respectively).

**S1 Table**

The effect of PTSD symptoms and fatigue on self-efficacy (*N*=527).

|  | *β* | *SE* | LLCI | ULCI |
| --- | --- | --- | --- | --- |
| Outcome: Self-efficacy |  |  |  |  |
| PTSD symptoms | -0.107* | 0.048 | -0.202 | -0.012 |
| Fatigue | -0.330*** | 0.048 | -0.424 | -0.235 |
| R^2^_adj_ | 0.186*** |  |  |  |
| F | 16.025 |  |  |  |

*Note*: All models are adjusted for age, gender, marital status, educational level, years of working and technical title.

^*^ *P* < 0.05

^***^ *P* < 0.001
